# Association between Primary Care Assessment Tool (PCAT) and Assessment of Chronic Illness Care (ACIC): a Brazilian cross-sectional study

**DOI:** 10.3389/fmed.2024.1374801

**Published:** 2024-11-04

**Authors:** Brenda Lorrana de Almeida Gomes, Renan Felipe Neves Mota, Renata Sant'ana Braga, Cynthia Assis de Barros Nunes, Rafael Alves Guimarães, Ana Paula dos Santos Rodrigues, Sandro Rogério Rodrigues Batista, Valéria Pagotto

**Affiliations:** ^1^School of Nursing, Federal University of Goiás, Goiânia, Brazil; ^2^Institute of Tropical Pathology and Public Health, Federal University of Goiás, Goiânia, Brazil; ^3^Health Department of Goiás State, Goiânia, Brazil; ^4^School of Medicine, Federal University of Goiás, Goiânia, Brazil; ^5^School of Medicine, University of Brasília, Brasília, Brazil

**Keywords:** Primary Health Care, noncommunicable diseases, chronic disease, quality of health care, process assessment, health care

## Abstract

**Aim:**

To analyze the association between Primary Health Care (PHC) performance and institutional ability to provide care for individuals with noncommunicable diseases (NCDs).

**Methods:**

Cross-sectional study conducted with primary care nurses and physicians in Brazil. The performance of PHC was assessed by using the Primary Care Assessment Tool (PCAT), whereas institutional ability for the care of people with NCDs was assessed through the Assessment of Chronic Illness Care (ACIC). Pearson correlation and multiple linear regression models were used to analyze the association between the PHC attributes measured in the PCAT (independent variables) and the ACIC dimensions (dependent variables).

**Results:**

In total, 308 health professionals −190 nurses (61.7%) and 118 physicians (38.3%)—at mean age 37.5 years and mean time of 6.5 years working in PHC participated of the study. On a scale of 0 to 10, the overall PCAT score was 6.74, while the ACIC score was 5.20. The PCAT score was High in only 58.8% of respondents (score ≥6.6). The ACIC scores showed basic institutional ability to care for people with NCDs. All ACIC dimensions have shown positive correlation to PCAT attributes, except for accessibility, continuity of care and care coordination.

**Conclusion:**

A positive association was found between PHC performance and institutional ability to care for people with NCDs. Results have evidenced the need of investing in PCH by providing technical, political, logistical and financial support to PHC units to improve PHC organization points and care for people with NCDs.

## 1 Introduction

Noncommunicable diseases (NCDs) comprise a set of long-lasting and slowly progressive diseases that require continuous and complex care (1). These diseases account for a global mortality burden, worldwide. Each year, 41 million people die from an NCDs, which is equivalent to 74% of all deaths in the world. Furthermore, 17 million people die from some NCDs before the age of 70 years, 86% in low and middle income countries (1). Cardiovascular diseases, cancer, chronic respiratory diseases and diabetes are the most frequent NCDs (2), whose increased rates in recent years are associated with population aging, as well as with increased modifiable risk factors, such as an unhealthy diet, physical inactivity and alcohol intake (3).

One of the targets set by the Sustainable Development Goals (SDGs) is “to reduce by one third premature mortality caused by noncommunicable diseases, by 2030, through prevention and treatment, as well as to promote mental health and wellbeing” (4). To do so, the process to tackle both NCDs and their risk factors must involve specific and fragmented actions that are far from the population's reality (5). Primary Health Care (PHC) is the care provision level enabling both organizing and integrating services to meet the needs of individuals with NCDs. Thus, PHC services that perform better may be more effective and qualified to care for people with NCDs (6).

Primary Care Assessment Tool (PCAT) is among the methodologies applied to assess both the incidence and extent of Brazilian PHC services' attributes (8). Studies have evidenced that PHC services in the country present high performance from health professionals' perspectives, their mean general scores range from 6.49 to 8.20 (9–14). Continuity of care stands out for showing the highest performance among other attributes, whereas accessibility presents the lowest performance (9–14).

Although PCAT is used to assess PHC performance, it is not capable of specifically assessing the quality of care provided for individuals with NCDs. On the other hand, the Assessment of Chronic Illness Care (ACIC) instrument enables healthcare institutions to provide care for chronic condition cases, based on six Chronic Care Model (CCM) elements (15). According to studies focused on investigating ACIC, health care organization, service delivery system design, and supported self-care were the components recording the best scores (16, 17). On the other hand, clinical decision support, the clinical information system, and articulation with the community were the components recording the worst scores (16, 17).

Although the PCAT and ACIC are applicable to PHC scenarios, to the best of our knowledge, the literature lacks studies focused on analyzing the association between them. However, some studies have evidenced that the better the PHC performance, the greater the number of practices and actions made available to individuals with NCDs (18, 19). Therefore, we herein hypothesize that the better the PHC services' performance, the higher the institutional ability to provide care for individuals with NCDs.

Considering the foregoing, the aim of the current study was to analyze the association between PHC performance and institutional ability to provide care for individuals with NCDs. This study can help strengthen the idea that well-performing PHC services substantiate the proposal to implement the CCM, since they can be used a reference to identify areas whose health- or community-care system requires improvements.

## 2 Material and methods

### 2.1 Study design

Cross-sectional and analytical study.

### 2.2 Setting

This study was included within a larger project study, “Training program focused on organizing and qualifying care provided for individuals with noncommunicable diseases in Goiás State's Primary Care Services,” which presented the objective, among others, of investigating the relationship between PHC performance and institutional ability to provide care for individuals with NCD.

The study was developed in Goiás state, located in the Central-West region of Brazil, whose territory comprises 246 cities and houses ~7,206,589 inhabitants (20). In total, 84 cities were selected based on the following criteria of the larger study: (i) obesity prevalence higher than the state's prevalence (28.6%), according to data provided by the Food and Nutrition Surveillance System (in Portuguese, *Sistema de Vigilância Alimentar e Nutricional*—SISVAN) from the Ministry of Health, to the detriment of data on hypertension and diabetes, since the aforementioned system encompasses the most updated and broadest datasets in Brazil (21), (ii) cities hosting an Expanded Family Health Center (in Portuguese, *Núcleo Ampliado de Saúde da Fam*í*lia*—NASF) that are composed of a multi professional team with the objective to development of matrix support and technical-pedagogical or clinical-assistance actions with collaborative activities with PHC teams (22), (iii) cities hosting a Health Academy Program (in Portuguese, *Academia da Saúde*), a Brazilian strategy that intends to support the health promotion by establishing public areas that are equipped with trained personnel and amenities to support physical exercise, leisure, and healthy living (23), and (iv) cities with professionals working with traditional peoples and communities (per example, *quilombolas* and indigenous peoples). These criteria, mainly (ii) and (iii), refer to health services associated with care provided for NCDs cases in Brazil.

### 2.3 Participants

The participants this study comprised nurses and physicians who worked in PHC services of all 84 selected cities in Goiás state. The Goiás State's counted on ~749 health teams at the beginning of this study. Nurses and physicians aged 18 or over, of both sexes, who actively worked in Basic Health Units (BHUs), and who filled out the questionnaire after three attempts of contact by the researchers' team, were included in the study. Professionals who did not respond after three contact attempts by the research team were excluded form study.

### 2.4 Study size

A sample size for simple correlation was carried out (24), considering a significance level of 5% (α = 0.05), statistical power of 90% (β = 0.10) and an expected correlation coefficient between the general PCAT and ACIC scores of, at least weak (*r* = 0.2), the minimum estimated sample was 259 health professionals. In the absence of studies that related the two instruments to support the sample calculation, we hypothesized that, at least, a weak correlation would be found.

### 2.5 Data collection and sampling

Data collection was carried out between September 2021 and June 2022. Our research team got in contact with management instances in all cities prior to data collection for to present the project and proposed schedule for data collection. At this time, all coordinators of the cities' PHC services were informed about the study by phone, and they provided both the email and phone number of PHC professionals who worked in the BHUs.

The sampling carried out was non-probabilistic for convenience. Research forms were sent to health professionals by text message and/or email. They were also contacted by phone to receive explanations about the project. Those who agreed to participate in the study completed the online form, which comprised sociodemographic, work-related, and professional qualification questions, as well as the PCAT—Brazilian extended version for professionals (7) and ACIC (15, 16) instruments. The research form of sociodemographic, work-related, and professional qualification questions was prepared by the researchers based on relevant previous literature that evaluated the performance of PHC in Brazil.

### 2.6 Variables

#### 2.6.1 Dependent variables

The dependent variables of the study were the PHC attributes. These were assessed by PCAT—Brazilian extended version for professionals (7), validated in Brazil. This instrument was created based on the health services quality assessment model proposed by Donabedian (25), which is based on measuring aspects related to the structure, process, and results of health services. The PCAT—Brazilian extended version for professionals can evaluate the PHC performance through the analysis of attributes. Attributes are defined as an interrelated and inseparable set of structuring elements present in health services and are used to evaluate PHC performance. Each one of these components comprises questions associated with the assessed attribute (7).

The PCAT—Brazilian extended version for professionals it presents 111 items and eight different attributes or dimensions, namely: first contact access (nine items), continuity of care (13 items), coordination of care—care integration (six items), coordination of care—information systems (eight items), comprehensiveness—services available (22 items), comprehensiveness—service delivery (18 items), family centeredness (14 items), and community orientation (21 items). First contact access refers to the accessibility and use of health services as a source of care for each new problem or new episode of the same health problem, except for emergencies and medical urgencies. Continuity of care refers to the existence of a continuous source of care and its use over time. Coordination presupposes some form of continuity of care by the same professional, through medical records or both, in addition to recognizing problems addressed in other services and integrating this care into the patient's overall care. It subdivides into two components (care integration and information systems). Comprehensiveness covers the set of services available and provided by PHC. These actions must offer comprehensive care, considering the biopsychosocial nature of the health-disease process, such as health promotion, disease prevention, treatment, and rehabilitation actions appropriate to the PHC context. It is divided into two components (services available and service delivery). Family centeredness refers to the fact that the assessment of individual needs for comprehensive care must consider the family context and its potential for care and, also, to threaten health, including the family approach to care. Community orientation refers to the recognition by services of community health needs through epidemiological data and direct contact with communities for joint planning and evaluation of services (7, 26).

The attributes first contact access, continuity of care, coordination of care—care integration, coordination of care—information systems, comprehensiveness—services available, comprehensiveness—service delivery is said to be essential attributes for evaluating PHC performance, while family centeredness, and community orientation encompass the derived attributes (7).

The calculation of PHC attribute scores followed the manual recommended by the Brazilian Ministry of Health (7). Response options of the PCAT—Brazilian extended version for professionals for each question are presented based on Likert scale ranging from 1 to 4 (1 = “Absolutely not,” 2 = “Likely not,” 3 = “likely yes” and 4 = “Yes, absolutely”), and on additional option 9, for “I do not know”/“I do not remember.” The higher the value attributed by the participant, the greater the extension or guidance for the PHC. One item (9 of the first contact access attribute) is formulated in such a way that the higher the value (response) assigned, the lower its orientation toward the PHC. Therefore, this item had its value inverted to: (value 4 = 1), (value 3 = 2), value (2 = 3) and (value 1 = 4). If for a participant, the sum of blank answers (“missing”) with “9” answers (“I don't know/don't remember”) reaches 50% or more of the total items of an attribute or dimension, the score should not be calculated for this attribute or dimension for this individual. The score for this attribute or dimension for this participant is left blank (“missing”). If for a participant, the sum of blank answers (“missing”) with answers “9” (“I don't know/don't remember”) is <50% of the total items of a component, the value “9” is transformed to the value “2” (“probably not”). This transformation is necessary to negatively highlight some characteristics of the health service that are not known to the interviewee. The crude scores for each of the attributes or dimensions are calculated by the arithmetic mean of the values of the responses to the items that make up each attribute or dimension, according to the formula below (7):


(1) $$\begin{array}{l} \text{Crude score}=\text{sum of attribute items}/\text{number of attribute items} \end{array}$$

Next, the scores were transformed into a scale from 0 to 10, using the following formula (7):


(2) $$\begin{array}{l} \text{Standard score }\left(\text{score }0-10\right)=\text{Crude escore}-1^{*}10/3 \end{array}$$

Finally, the essential scores were calculated, using the arithmetic mean of the essential attributes (first contact access, continuity of care, coordination of care—care integration, coordination of care—information systems, comprehensiveness—services available, comprehensiveness—service delivery) and derived, through the arithmetic mean of the derived attributes (family centeredness, and community orientation). The overall score was calculated by the arithmetic mean of the score of essential and derived attributes (7).

A standard score of zero indicates a lower presence and extent of PHC attributes, while a score of 10 suggests a greater presence and extent. The final classification defined PHC performance as “strong” (for final scores ≥6.6) and “weak” (for values <6.6) for each attribute and scores. Strong scores have evidenced both the incidence and extent of primary care attributes, and it indicated services better oriented to this health care level (7).

More details on the PCAT—Brazilian extended version for professionals' calculation method is previously published (7).

#### 2.6.2 Independents variables

The domains of institutional ability to provide care for chronic conditions and diseases, were considered independent variables and were measured by the ACIC instrument. The objective of this instrument is to evaluate the institutional capacity to care for people with chronic conditions based on elements of the MCC, through the perception of health professionals. It should be used by health professionals to identify areas of greater fragility with the aim of qualifying care for people with chronic conditions (15). The instrument was translated and cross-culturally adapted into Portuguese (27).

It comprises 36 questions that cover seven CCM dimensions, namely: health care organization (six items), connection with the community (four items), supported self-care (four items), clinical decision support (four items), service provision system design (six items), clinical information system (six items), and model integration (six items). The health care organization is based on the concept that care for chronic conditions is more effective if the health system is oriented and allows greater emphasis on care for chronic diseases. The connection with the community is because the articulation between the health system and community resources plays a fundamental role in the management of chronic conditions. Self-care support can help individuals with chronic conditions and their families to deal with the challenges and conditions of living with and treating chronic conditions, enabling the reduction of complications and symptoms. Clinical decision support is because the management of chronic conditions ensures that healthcare professionals have access to evidence-based information to support clinical decisions. Service provision system design refers to the fact that effective management of care for chronic conditions involves adding interventions to a system focused on the care of acute conditions. The clinical information system is because useful and timely information, personalized for individual users and chronic condition group populations, is a critical aspect of effective care models. Model integration refers to the fact that effective health systems integrate and combine all elements of the model, for example, associating self-care goals with data from health information systems or associating local policies with activities in users' therapeutic plans (27).

Each ACIC item has a scale from 0 to 11, where 0 represents the lowest score, that is, a place with limited resources or structures, and 11, the highest score, a place with resources and excellent structure for care for chronic conditions. The scores for each dimension are calculated using the arithmetic mean using the following formula (27):


(3) $$\begin{array}{l} \text{Score}=\text{sum of dimension items}/\text{number of dimension items} \end{array}$$

A general score is also calculated by summing the scores for each dimension divided by the number of dimensions (7) (27).

A lower mean dimension and overall score indicates lower institutional capacity to care for people with NCDs and a higher mean indicates greater capacity. The scores for each dimension and general generate a categorization into four levels that enable the interpretation of the results: scores ranging from 0 to 2 referred to the limited ability of services to provide care for chronic health conditions, scores ranging from 3 to 5 referred to the basic ability of services to provide care for chronic health condition cases, scores ranging from 6 to 8 referred to the reasonable ability of services to provide care for chronic health condition cases, and scores ranging from 9 to 11 referred to the strong ability to provide care for NCDs cases (27).

More details on the ACIC calculation method is previously published (27).

#### 2.6.3 Covariables

The following study covariates were considered: age (in years), sex (male, female), race (white, mixed-race, black and Asian), profession category (nurses, physicians), time since training in the profession (years), training institution type (private and public), post-graduation certificate (yes, no), time in job position (in years), time working in PHC (in years) and, employment relationship (statutory/fixed, hired, residence scholarship).

### 2.7 Statistical analysis

Data analysis was carried out in R language (version 4.0.2, R Foundation for Statistical Computing, Vienna, Austria) (28).

The Anderson-Darling test was used to analyze the normality of quantitative variables. Descriptive statistics of the demographic, professional characteristics and PCAT and ACIC variables of all participants were performed. Quantitative variables were presented as mean, standard deviation (SD), median, 25th percentile (P25), 75th percentile (P75), minimum and maximum value, since all variables showed no Gaussian distribution. Additionally, 95% confidence intervals (95% CI) for mean were presented for PCAT and ACIC scores. Qualitative variables were presented as absolute (*n*) and relative frequency (%). Furthermore, a 95% CI for proportion was used for the percentages of high scores (≥6.6) assessed on the PCAT and for each classification of ACIC scores (limited ability, basic ability, reasonable ability and strong ability).

Pearson's correlation coefficient (*r*) was used to analyze the bivariate relationship between the scores of each PCAT attribute and ACIC dimensions, as well as the relationship between their overall scores. A parametric analysis was chosen even in the absence of normality, due to the sufficiently large sample, due to the Central Limit Theorem (29). The correlation was classified as 0.00–0.19—very weak, 0.20–0.39—weak, 0.40–0.59—moderate, 0.60–0.79—strong and, 0.80–1.00—very strong (30).

Finally, multiple linear regression models were used to investigate association between ACIC (dependent variables) and PCAT scores (dependent variables). Each model was adjusted for covariates. Each ACIC attribute, used as independent variables, was modeled in separate regressions due to the high correlation between the attributes, minimizing the potential for multicollinearity. This was confirmed using the Pearson correlation matrix between the independent variables. For example, when the “first contact access” attribute score was used as the dependent variable, eight models were adjusted (each containing the seven dimensions and the overall ACIC score).

The magnitude of the assessed association was expressed as regression coefficient (β) and 95% CI. Variables recording *p*-value <0.05 were considered statistically significant.

### 2.8 Ethical aspects

The current study was approved by the Research Ethics Committee of the Clinical Hospital of Federal University of Goiás, protocol number 6.037.327/2020. Online consent was obtained for all participants before the individual completed the interview.

## 3 Results

In total, 308 health professionals −190 nurses (61.7%) and 118 physicians (38.3%)—participated in this study. They presented a mean age of 37.5 years (SD = 9.6) and the majority were female (82.8%). Regarding race, 43.5% self-declared white, 38.0% mixed-race, 6.2% black, and 2.3% Asian. As for training, the average time since graduation was 9.5 years (SD = 8.1) and the majority (71.8%) attended college at private institutions. A total of 76.9% had a postgraduate course (specialization, residency, master's degree, and/or doctorate). The average time in the current working position was 4.7 years (SD = 5.4) and the average time working in PHC was 6.5 years (SD = 6.3). The majority (58.4%) of participants had a contract job (Table 1).

**Table 1:** Demographic and professional features of the sample.

**Variables**	**Total (*n* = 308)**
**Age (years)**	
Mean (SD)	37.5 (9.6)
Median (P25–P75)	35.5 (31.0–43.0)
Minimum–Maximum	22.0–75.0
**Sex**, ***n*** **(%)**	
Female	255 (82.8)
Male	53 (17.2)
**Race**, ***n*** **(%)**	
White	134 (43.5)
Mixed-race	148 (48)
Black	19 (6.2)
Asian	7 (2.3)
**Professional category**, ***n*** **(%)**	
Nurse	190 (61.7%)
Physician	118 (38.3%)
**Time since training in the profession (years)**	
Mean (SD)	9.5 (8.1)
Median (P25–P75)	8.0 (3.0–13.0)
Minimum–Maximum	0.2–46.0
**Training institution type**, ***n*** **(%)**	
Public	87 (28.2)
Private	221 (71.8)
**Post-graduation certificate**, ***n*** **(%)**	
Yes	237 (76.9)
No	71 (23.1)
**Time in job position (years)**	
Mean (SD)	4.7 (5.4)
Median (P25–P75)	3 (0.9–7.0)
Minimum–Maximum	0.1–27.0
**Time working in PHC (years)**	
Mean (SD)	6.5 (6.3)
Median (P25–P75)	4.0 (1.8–10.0)
Minimum–Maximum	0.1–27.0
**Employment relationship**, ***n*** **(%)**	
Statutory/fixed	108 (35.1)
Hired	180 (58.4)
Residency scholarship	20 (6.5)

BHUs, Basic Health Units; P25, 25th percentile; P75, 75th percentile; PHC, Primary Health Care; SD, Standard deviation.

The analysis of PHC performance data using the PCAT for professionals showed that the average scores showed wide variation between attributes. The lowest mean was found for the “first contact access” attribute (Mean = 5.09, SD = 1.68). The highest means were observed for the attributes “comprehensiveness—services available” (Mean = 7.00, SD = 1.37), “family centeredness” (Mean = 7.13, SD = 1.78) and “comprehensiveness—service delivery” (Mean = 8.01, SD = 1.56). The derived and general scores had means of 6.74 (SD = 1.05) and 6.81 (SD = 1.47), respectively. The overall score had a mean of 6.77 (SD = 1.18). The percentages of high scores also varied between attributes. The attribute “first contact access” presented a small percentage of high scores (18.5%), indicating less presence and extension of this attribute, while the attribute “comprehensiveness—service delivery” presented the highest percentage (80.2%), suggesting greater presence and extension of this attribute in PHC in the study sample (Table 2).

**Table 2:** Descriptive statistics of the attributes and scores of the Primary Care Assessment Tool (PCAT).

**PHC attributes and scores**	**Mean**	**95% CI**	**SD**	**Median**	**Minimum**	**Maximum**	**P25**	**P75**	**Proportion high score (**≥**6.6)**	
									**%**	**95% CI**
First contact access	5.09	4.90–5.28	1.68	4.81	1.48	10.00	4.07	5.93	18.5	14.5–23.3
Continuity of care	6.80	6.67–5.95	1.32	6.67	3.59	10.00	5.90	7.69	55.5	59.9–61.0
Coordination of care—care integration	6.86	6.70–7.02	1.45	6.67	3.33	10.00	5.56	7.78	62.0	56.0–67.3
Coordination of care—information systems	6.66	6.50–6.82	1.43	6.67	2.08	10.00	5.83	7.50	56.4	50.9–62.0
Comprehensiveness—services available	7.00	6.84–7.15	1.37	7.12	3.03	9.39	6.06	7.88	64.3	57.8–68.5
Comprehensiveness—service delivery	8.01	7.83–8.18	1.56	8.15	2.22	10.00	6.85	9.44	80.2	75.3–84.3
Family centeredness	7.13	6.93–7.32	1.78	6.90	0.71	10.00	5.95	8.57	64.0	58.4–69.2
Community orientation	6.48	6.30–6.66	1.63	6.75	0.16	9.05	5.67	7.62	55.8	50.2–61.3
Essential score	6.74	6.62–6.85	1.05	6.73	3.10	9.81	6.12	7.42	54.9	49.2–60.3
Derived score	6.81	6.64–6.85	1.47	6.94	1.55	9.52	5.95	7.75	58.4	52.2–63.8
General score	6.77	6.64–6.90	1.18	6.88	2.32	9.59	6.03	7.55	58.8	53.2–64.2

95% CI, 95% Confidence interval; P25, 25th percentile; P75, 75th percentile; SD, standard deviation.

The ACIC analysis showed that the scores also varied between the dimensions of institutional ability to provide care for people with NCDs. The lowest averages were found for the “clinical decision support” (Mean = 4.87, SD = 2.61) and “model integration” (Mean = 4.83, SD = 2.64) dimensions, while the highest was observed in the “service provision system design” dimension (Mean = 5.92, SD = 2.64). The overall score presented a mean score of 5.20 (SD = 2.38). All dimensions and general score showed basic ability to care for people with NCDs (Table 3).

**Table 3:** Descriptive statistics of the dimensions of the Assessment of Chronic Illness Care (ACIC).

**ACIC dimensions**	**Mean**	**95% CI**	**SD**	**Median**	**Minimum**	**Maximum**	**P25**	**P75**	**Classification**
Health care organization	5.25	4.98–5.53	2.48	5.17	1.00	11.00	3.33	7.17	Basic ability
Connection to the community	5.16	4.88–5.44	2.48	5.00	1.00	11.00	3.00	7.00	Basic ability
Supported self-care	5.28	4.99–5.56	2.56	5.25	1.00	11.00	3.00	7.25	Basic ability
Clinical decision support	4.87	4.58–5.17	2.61	4.50	1.00	11.00	2.75	7.00	Basic ability
Service provision system design	5.92	5.62–6.22	2.64	6.00	1.00	11.00	3.63	8.17	Basic ability
Clinical information system	5.06	4.76–5.36	2.66	4.67	1.00	11.00	2.83	7.17	Basic ability
Model integration	4.83	4.54–5.13	2.64	4.42	1.00	11.00	2.67	6.67	Basic ability
General	5.20	4.93–5.46	2.38	4.90	1.00	10.86	3.31	7.05	Basic ability

95% CI, 95% Confidence interval; P25, 25th percentile; P75, 75th percentile; SD, Standard deviation.

**Figure 1:**
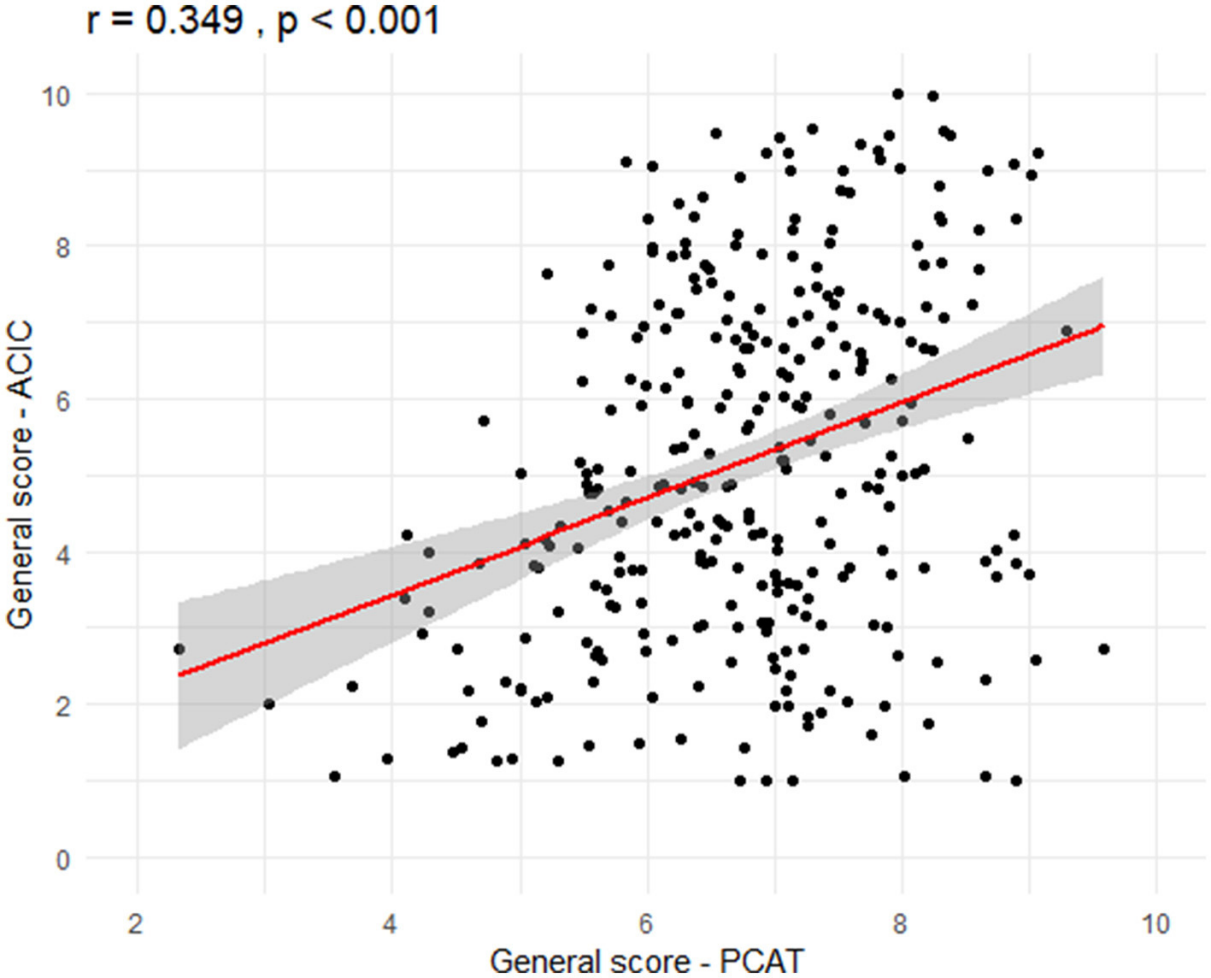
Correlation between overall scores of the Primary Care Assessment Tool (PCAT) and Assessment of Chronic Illness Care (ACIC).

Person's correlation analysis between the general score of the ACIC and the PCAT showed a weak positive correlation (*r* = 0.349, *p* < 0.001; Figure 1). This suggests, albeit weakly, an increase in the general performance of APH with the increase in institutional ability to provide care for people with NCDs. All PCAT attributes have shown positive and statistically significant correlation to ACIC dimensions, except for “health care organization” dimension and attributes “first contact access” (*r* = 0.444, *p* = 0.442), “continuity of care” (*r* = 0.056, *p* = 0.328) and “coordination of care—care integration” (*r* = 0.100, *p* = 0.079) and the “service provision system design” dimension and attribute “first contact access” (*r* = 0.093, *p* = 0.102). Most statistically significant correlations were weak (Table 4).

**Table 4:** Correlation between attributes and scores of the Primary Care Assessment Tool (PCAT) and dimensions of the Assessment of Chronic Illness Care (ACIC).

**PHC attributes and scores**	**ACIC dimensions**															
	**Health care organization**		**Connection to the community**		**Supported self-care**		**Clinical decision support**		**Service provision system design**		**Clinical information system**		**Model integration**		**General**	
	***r***	***p***	***r***	***p***	***r***	***p***	***r***	***p***	***r***	***p***	***r***	***p***	***r***	***p***	***r***	***p***
First contact access	0.044	0.442	0.159	0.005	0.116	0.042	0.137	0.016	0.093	0.102	0.1780	0.002	0.216	<0.001	0.147	0.010
Continuity of care	0.056	0.328	0.110	0.053	0.113	0.048	0.115	0.044	0.115	0.044	0.117	0.040	0.146	0.010	0.124	0.029
Coordination of care—care integration	0.150	0.008	0.185	0.001	0.209	<0.001	0.219	<0.001	0.184	0.001	0.203	<0.001	0.241	<0.001	0.216	<0.001
Coordination of care—information systems	0.100	0.079	0.139	0.015	0.164	0.004	0.187	<0.001	0.1434	0.012	0.241	<0.001	0.269	<0.001	0.194	<0.001
Comprehensiveness—services available	0.192	<0.001	0.237	<0.001	0.238	<0.001	0.247	<0.001	0.205	<0.001	0.264	<0.001	0.321	<0.001	0.264	<0.001
Comprehensiveness—service delivery	0.205	<0.001	0.209	<0.001	0.226	<0.001	0.181	0.001	0.193	<0.001	0.223	<0.001	0.276	<0.001	0.234	<0.001
Family centeredness	0.155	0.007	0.161	0.005	0.231	<0.001	0.166	0.004	0.139	0.015	0.174	0.002	0.231	<0.001	0.194	<0.001
Community orientation	0.329	<0.001	0.398	<0.001	0.378	<0.001	0.374	<0.001	0.372	<0.001	0.412	<0.001	0.467	<0.001	0.422	<0.001
Essential score	0.173	0.002	0.242	<0.001	0.254	<0.001	0.251	<0.001	0.216	<0.001	0.286	<0.001	0.342	<0.001	0.245	<0.001
Derived score	0.275	<0.001	0.317	<0.001	0.3478	<0.001	0.305	<0.001	0.289	<0.001	0.332	<0.001	0.397	<0.001	0.350	<0.001
General score	0.248	<0.001	0.305	<0.001	0.330	<0.001	0.301	<0.001	0.276	<0.001	0.334	<0.001	0.399	<0.001	0.340	<0.001

r, Pearson correlation coefficient.

**Figure 2:**
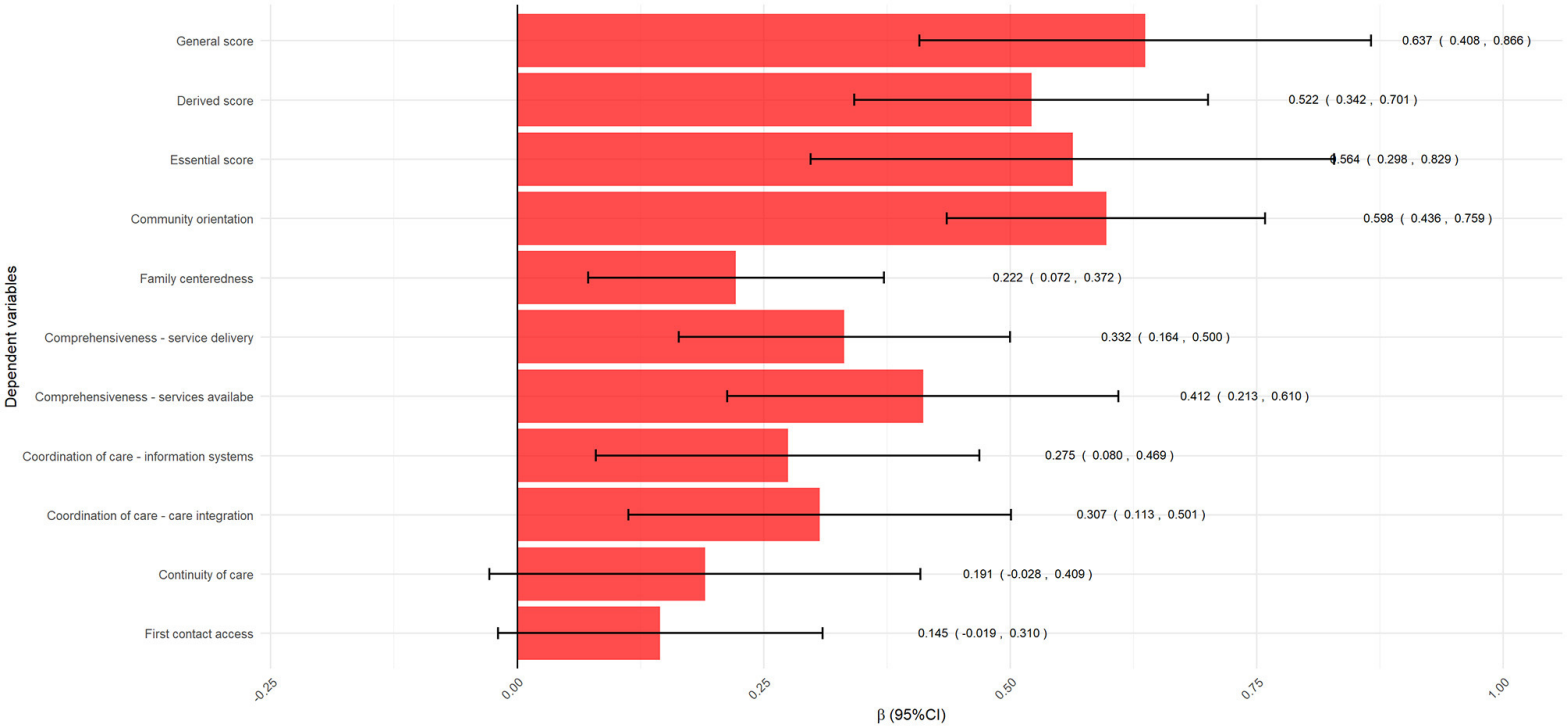
Linear multiple regression results between general score of the Assessment of Chronic Illness Care (ACIC) and attributes and scores of the Primary Care Assessment Tool (PCAT).

The multiple linear regression model showed a positive association between the general score of the ACIC, and all attributes and scores measured in the PCAT except for the attributes “first contact access” (β = 0.145, 95% CI: −0.019, 0.310) and “continuity of care” (β = 0.191, 95% CI: −0.028, 0.049). This indicates that, with each increase in the institutional ability to provide care for people with NCDs, the extent of PHC performance increases in all attributes and scores evaluated (Figure 2).

The Table 5 presents the β regression coefficients and their 95% confidence intervals for different dimensions of the ACIC and attributes and scores of the PCAT. Results indicate significant positive associations (indicated by asterisks and double asterisks) between the different components of the ACIC and PCAT. For example, model integration has strong positive associations with all attributes and scores of the PCAT, highlighting the importance of these factors in effective integration between the integration model for care for people with NCD and community guidance. Different strengths and positive associations were verified for most of the regressions carried out between the other components of the ACIC and PCAT, showing the influence of the institutional ability to provide care for people with NCDs on the PHC performance.

**Table 5:** Linear regression analysis between attributes and scores of the Primary Care Assessment Tool (PCAT) and dimensions of the Assessment of Chronic Illness Care (ACIC).

**PHC attributes and scores**	**ACIC dimensions**						
	**Health care organization**	**Connection to the community**	**Supported self-care**	**Clinical decision support**	**Service provision system design**	**Clinical information system**	**Model integration**
	β **(95% CI)**	β **(95% CI)**	β **(95% CI)**	β **(95% CI)**	β **(95% CI)**	β **(95% CI)**	β **(95% CI)**
First contact access	−0.001 (−0.174, 0.172)	0.181^*^ (0.009, 0.354)	0.111 (−0.068, 0.290)	0.148 (−0.031, 0.328)	0.057 (−0.125, 0.240)	0.233^*^ (0.048, 0.418)	0.288^**^ (0.107, 0.469)
Continuity of care	0.050 (−0.179, 0.280)	0.180 (−0.049, 0.409)	0.264^*^ (0.028, 0.500)	0.201 (−0.037, 0.439)	0.177 (−0.064, 0.418)	0.189 (−0.057, 0.436)	0.273^*^ (0.031, 0.515)
Coordination of care—care integration	0.217^*^ (0.012, 0.421)	0.271^**^ (0.067, 0.475)	0.335^**^ (0.125, 0.545)	0.336^**^ (0.125, 0.547)	0.252^*^ (0.037, 0.467)	0.336^**^ (0.118, 0.555)	0.406^***^ (0.192, 0.620)
Coordination of care—information systems	0.135 (−0.070, 0.340)	0.200 (−0.005, 0.405)	0.257^*^ (0.046, 0.469)	0.278^*^ (0.067, 0.490)	0.192 (−0.023, 0.408)	0.406^***^ (0.189, 0.623)	0.457^***^ (0.245, 0.670)
Comprehensiveness—services available	0.310^**^ (0.100, 0.520)	0.378^***^ (0.169, 0.587)	0.397^***^ (0.180, 0.613)	0.423^***^ (0.207, 0.639)	0.332^**^ (0.111, 0.553)	0.469^***^ (0.246, 0.692)	0.576^***^ (0.360, 0.793)
Comprehensiveness—service delivery	0.303^***^ (0.127, 0.480)	0.302^***^ (0.125, 0.479)	0.347^***^ (0.164, 0.529)	0.273^**^ (0.089, 0.458)	0.296^**^ (0.109, 0.482)	0.362^***^ (0.172, 0.551)	0.445^***^ (0.261, 0.630)
Family centeredness	0.183^*^ (0.026, 0.341)	0.187^*^ (0.030, 0.345)	0.304^***^ (0.143, 0.465)	0.196^*^ (0.032, 0.360)	0.155 (−0.011, 0.321)	0.224^**^ (0.054, 0.393)	0.308^***^ (0.142, 0.472)
Community orientation	0.484^***^ (0.309, 0.658)	0.584^***^ (0.414, 0.755)	0.582^***^ (0.404, 0.760)	0.561^***^ (0.382, 0.741)	0.558^***^ (0.376, 0.740)	0.660^***^ (0.477, 0.842)	0.758^***^ (0.584, 0.932)
Essential score	0.345^*^ (0.062, 0.627)	0.519^***^ (0.239, 0.799)	0.572^***^ (0.284, 0.861)	0.557^***^ (0.267, 0.847)	0.437^**^ (0.140, 0.733)	0.684^***^ (0.386, 0.982)	0.836^***^ (0.549, 1.124)
Derived score	0.425^***^ (0.233, 0.616)	0.486^***^ (0.296, 0.677)	0.577^***^ (0.382, 0.771)	0.480^***^ (0.281, 0.679)	0.446^***^ (0.244, 0.648)	0.559^***^ (0.355, 0.763)	0.681^***^ (0.486, 0.876)
General score	0.475^***^ (0.220, 0.720)	0.592^***^ (0.350, 0.834)	0.685^***^ (0.437, 0.933)	0.601^***^ (0.349, 0.853)	0.527^***^ (0.270, 0.785)	0.714^***^ (0.456, 0.972)	0.870^***^ (0.623, 1.118)

^***^p <0.001.

^**^p <0.01.

^*^p <0.05.

95% CI, 95% confidence interval; β, regression coefficient.

## 4 Discussion

Results in the current study have shown positive association between institutional ability to provide care for individuals with NCDs and PHC performance. These results show that an institutional capacity for people with NCDs increases the performance of the PHC, which can influence the provision of qualified services to people with NCDs in this health care scenario. To the best of our knowledge, association between ACIC and PCAT had not been analyzed in previous studies. Therefore, based on the understanding that service's organization guide this PHC, such an association highlights PHC's relevance in providing care for individuals with NCDs.

The current study observed strong scores of PHC services, with an overall average of 6.73. This finding had been previously observed in other studies conducted in different places in Brazil (9–12, 31). This result is also higher than the general score estimated for the Brazilian population (5.9) (32), indicating an important and positive finding for Brazilian PHC in the context under study. It is considered that the current research was carried out during the second COVID-19 pandemic wave, when the entire Brazilian healthcare service network, including PHC units, was reorganized to deal with this health crisis. However, these results are lower than those found in other studies carried out in Brazil in the perception of health professionals (12, 33), indicating that there are possibilities for improvement to achieve better PHC performance. Still, in relation to our study population, it is necessary to highlight that it is composed of health professionals and, in this situation, is expected to have more favorable perceptions of professionals than users and, therefore, the PCAT score found in this study may be underrated (33). We also found a lower score for the first contact access dimension, indicating greater limitations in access to PHC health services and the need to expand this attribute to achieve universal health coverage. The lowest score for this attribute when compared to the others was also reported in previous studies conducted in Brazil (12, 33).

We found that the institutional ability to provide care for individuals with NCDs was classified as basic, from physicians and nurses' perspective for all dimensions and overall ACIC score. This classification may be associated, among other aspects, with little interest of organizational leadership in changing or implementing CCM in PHC, weak relationship between health units and the community, population's poor knowledge about its clinical conditions and self-management of comorbidities, inefficient continuous education system capable of hindering clinical decision-making, and difficulty in monitoring and having access to users' information due to faulty information systems. However, studies are needed to confirm these hypotheses. There is limited literature that has evaluated ACIC scores in PHC. However, studies have shown scores ranging from 3.15 to 8.98 (15–17, 34). This difference is possibly due to differences in the characteristics of the study populations, in addition to the different degrees of attention to caring for people with NCDs in different settings.

The “service provision system design” recorded the highest score among ACIC dimensions, and it was consistent with previous research (17). This dimension refers to system organization and aims at providing care for individuals with NCDs. This organization process involves teamwork, health teams' leadership, scheduling, monitoring and scheduled-NCDs-care system, and continuous care, indicating improvements in the reorganization of the care model for NCDs (27). Previous study have evidenced that changes in PHC services, such as appointing periodic consultations (35) and developing chronic care-management programs for diabetes cases, resulted in better health outcomes (36). There is evidence that organizational changes, such as filling electronic medical records, makes patient monitoring easier. In addition, supporting professionals' continuous education, mostly on diabetes self-management, has positive impact on the coordination of care provided for patients with diabetes (37). Although this dimension presented the best performance in the current study, its score corresponded to basic ability. Therefore, it is important improving the service delivery system focused on individuals with NCDs based on strategies, such as improving teamwork, providing group and distance care, and improving the scheduled care system.

The clinical support decision dimension was responsible for the lowest scores. The first dimension refers to professionals' access to information available to help decision-making processes focused on providing care for individuals with CC, including continuous education, evidence-based clinical guidelines and experts' involvement in decision-making (27). These low scores may disclose gaps in continuous health education in PHC, which is essential to help professionals to develop both the skills and autonomy necessary to provide proper care for individuals with NCDs (38). According to literature review, difficulty of being released, work overload and lack of planning are among factors hindering the implementation of continuous education strategies, and it results in initiatives that, oftentimes, are not consistent with PHC professionals' reality (39). Therefore, it is necessary identifying weak points to enable initiatives to be carried out in an assertive and powerful manner to favor the teaching-learning process. Among the initiatives, one finds PHC professionals' integration to specialized care, providing incentives to professionals and creating reminder and feedback systems to help decision-making processes (5).

Supported self-care also was classified as basic. This dimension refers to helping individuals with NCDs, and their families, to self-manage their health to help mitigating disease complications and symptoms (5, 22). This support can be provided through care–plan development, with emphasis on the central role played by the person with NCDs, based on strategies to teach how to self-assess health status and to embrace one's concerns (7). Previous study has evidenced that interventions focused on supporting self-care, such as independent monitoring of symptoms, self-treatment in response to worsened symptoms, strategies focused on managing stress and worries, and progress monitoring, had positive impact on both health outcomes and quality of life of patients with NCDs (40). It is necessary developing strategies to increase patients' participation in decision-making and in care provided for their clinical condition.

Clinical information system also equally was classified as basic. The clinical information system dimension refers to organizing information about users with NCDs to make healthcare provision easier. It can be done through electronic medical records and adherence to a system to enable health professionals in the team and users to share clinical information, as well as through warns and feedback provided for healthcare teams (5, 27). Systematic review pointed out that technologies, such as sensors and wearables, have been used to remotely monitor clinical conditions in the PHC context and to help maintaining information about patients. However, difficulties in integrating new technological systems to systems currently operating in PHC units hinder professionals' work process (41). Thus, it is necessary structuring an effective clinical information system to enable continuously monitoring patients' clinical condition and to help health professionals during healthcare provision processes.

The score observed for dimensions “articulation with the community” and “model integration” was classified as basic. The score herein recorded for “articulation with the community” has indicated that the connection between community resources and health services may not provide adequate support to better cope with NCDs. It is known that better coordinated services tend to have better health outcomes, given the role played by community resources in the process to promote, prevent and cope with NCDs (42). Therefore, it is essential knowing community resources (social facilities), seeking partnerships with community leaders and encouraging patients' participation in community actions to help better managing NCDs (22). The low score in the dimension “model integration” toward difficulty in integrating the previously addressed dimensions, i.e., in integrating clinical guidelines for clinical conditions, information systems, community programs and target monitoring, for example. Effective health systems must integrate all CCM elements, and their integration level reflects the way health professionals provide care for patients with NCDs.

One of the main results of the study is the positive association between the institutional ability to care for people with NCDs in different dimensions and the multiple attributes that measure the performance of the PHC. We did not identify studies that analyzed the relationship between ACIC and PCAT, as mentioned previously. However, the results show that the effective organization of services according to a model for caring for people with NCDs influences the performance of the PHC, indicating the urgent need to reorganize services aimed at specific care for the population of people with NCDs. The results also indicate that the “model integration” dimension has greater influence (strength) on the PCAT attribute scores, suggesting the importance of integrated care for PHC performance in the Brazilian context.

This study had some limitations. The cross-sectional nature of the study did not enable defining temporality between independent and dependent variables (ACIC and PCAT scores, respectively), therefore, it was not possible to determine causality. The data is self-reported, subject to memory bias and participant response. The risk of selection bias may have occurred due to the convenience sample and online recruitment, therefore, we cannot generalize the study results to the entire population of PHC nurses and physicians. Previous studies that have applied these instruments did so in a self-administered and face-to-face manner. It is possible that the characteristics of professionals and their perceptions are different from those who did not have access to the online form to respond. Although the current study was conducted remotely, it followed all team training procedures and provided information about the research to participants. The response rate was relatively low, which may have led to an underestimation of the scores and associations found. Again, due to this limitation, the generalization of the results is limited. Another limitation was the data collection period, which took place during the second wave of the COVID-19 pandemic. Because this study focused on analyzing PHC services' performance, professionals' perceptions about each dimension assessed in the two adopted instruments may have been influenced by changes in PHC resulting from this health crisis. We also highlight the need for caution when interpreting the results, because although the majority of scores obtained a positive result, evidence of the quality of primary care with users points the opposite, which could be counterintuitive to the public reality of Brazilian PHC. Finally, another limitation was the lack of collection of user data for comparison with the results found in the perception of health professionals.

This study has evidenced an association between institutional ability to care for individuals with NCDs and PHC performance. Concerning the institutional ability to provide care for individuals with NCDs, the organizational capacity of the CCM influences the performance of the PHC, potentially influencing the quality of care provided for this population. The present research has portrayed both the potential and weaknesses of PHC and the care offered to patients with NCDs in these services. Thus, it can be used to substantiate strategies to improve PHC services and provide care for patients with chronic conditions. It is worth highlighting the importance of conducting further studies to help better understand the association between institutional ability to provide care for individuals with NCDs and PHC performance in other Brazilian regions. These studies can help strengthen PHC and implement CCM in healthcare units, also positively impacting the care for individuals with NCDs.

## Data Availability

The raw data supporting the conclusions of this article will be made available by the authors, without undue reservation.
